# Survival of HT29 Cancer Cells Is Affected by IGF1R Inhibition *via* Modulation of Self-DNA-Triggered TLR9 Signaling and the Autophagy Response

**DOI:** 10.3389/pore.2022.1610322

**Published:** 2022-05-16

**Authors:** Ferenc Sipos, Bettina Bohusné Barta, Ágnes Simon, Lőrinc Nagy, Titanilla Dankó, Regina Eszter Raffay, Gábor Petővári, Viktória Zsiros, Barnabás Wichmann, Anna Sebestyén, Györgyi Műzes

**Affiliations:** ^1^ Department of Internal Medicine and Hematology, Semmelweis University, Budapest, Hungary; ^2^ Department of Pathology and Experimental Cancer Research, Semmelweis University, Budapest, Hungary; ^3^ Department of Anatomy, Histology and Embryology, Semmelweis University, Budapest, Hungary; ^4^ Lufthansa Systems Hungária, Budapest, Hungary

**Keywords:** autophagy, CD133, IGF1R, TLR9, HT29 cancer cell, self-DNA

## Abstract

**Purpose:** In HT29 colon cancer cells, a close interplay between self-DNA-induced TLR9 signaling and autophagy response was found, with remarkable effects on cell survival and differentiation. IGF1R activation drives the development and malignant progression of colorectal cancer. IGF1R inhibition displays a controversial effect on autophagy. The interrelated roles of IGF1R inhibition and TLR9/autophagy signaling in HT29 cancer cells have not yet been clarified. In our study, we aimed to investigate the complex interplay of IGF1R inhibition and TLR9/autophagy signaling in HT29 cells.

**Methods:** HT29 cells were incubated with tumor-originated self-DNA with or without inhibitors of IGF1R (picropodophyllin), autophagy (chloroquine), and TLR9 (ODN2088), respectively. Cell proliferation and metabolic activity measurements, direct cell counting, NanoString and Taqman gene expression analyses, immunocytochemistry, WES Simple Western blot, and transmission electron microscopy investigations were performed.

**Results:** The concomitant use of tumor-derived self-DNA and IGF1R inhibitors displays anti-proliferative potential, which can be reversed by parallel TLR9 signaling inhibition. The distinct effects of picropodophyllin, ODN2088, and chloroquine *per se* or in combination on HT29 cell proliferation and autophagy suggest that either the IGF1R-associated or non-associated autophagy machinery is “Janus-faced” regarding its actions on cell proliferation. Autophagy, induced by different combinations of self-DNA and inhibitors is not sufficient to rescue HT29 cells from death but results in the survival of some CD133-positive stem-like HT29 cells.

**Conclusion:** The creation of new types of combined IGF1R, autophagy, and/or TLR9 signaling inhibitors would play a significant role in the development of more personalized anti-tumor therapies for colorectal cancer.

## Introduction

Insulin-like growth factor 1 receptor (IGF1R), a transmembrane protein belonging to the receptor tyrosine kinase family, consists of two subunits (i.e., IGF1R-α and IGF1R-β). IGF1R ligands include insulin and insulin-like growth factors (IGF)-1 and -2. Upon ligand stimulation, the auto-phosphorylated IGF1R-β phosphorylates adaptor proteins (e.g., IRS1/2, SHC, 14-3-3); afterwards, it activates downstream signaling pathways, like PI3K/AKT, JAK/STAT, Src, FAK and RAS/MAPK, finally modulating gene expressions related to apoptosis or cell proliferation [1, 2]. Within physiological circumstances, IGF1R is frequently expressed in various tissues, serving multiple functions in growth, development, and feeding [3].

IGF1R activation in tumors can promote tumorigenesis, maintain the transformed phenotype, promote cancer progression, stimulate cell migration, epithelial-mesenchymal transformation, and chemotherapy resistance [4, 5]. In colorectal cancer (CRC), IGF1R gene and protein expression levels are usually elevated in cancerous tissues as compared to adjacent normal tissues [6]. Elevated IGF1R expression is associated with poor prognosis in CRC [7]. Given the significant roles of IGF1R in tumor development and progression, inhibition of IGF1R activity has been suggested as a therapeutic strategy for many malignancies [8]. Although several anti-IGF1R monoclonal antibodies and small-molecule inhibitors have been produced, and these inhibitors display potent anti-tumor effects in preclinical models [8], clinical trials of these agents are mostly disappointing in unselected cancer patients, suggesting the existence of mechanisms to antagonize IGF1R inhibition in tumor cells.

Autophagy is an evolutionally conserved proteolytic process including lysosomal degradation and recycling of impaired cellular components and energy to maintain homeostasis [9]. In preclinical studies, protective autophagy blockade has been applied simultaneously with either chemotherapies or targeted therapies to optimize their efficacy in different cancers [10]. The connection of the IGF1R signaling pathway to the autophagy machinery is rather complicated [11]. Furthermore, IGF1R inhibition has been shown to have different effects (i.e., inhibitory or stimulatory) on autophagy in cancer cells [12–14]. Recently, it has been found that the combined use of autophagy-disrupting agents can enhance the therapeutic efficacy of IGF1R inhibitors in triple-negative breast cancers and may therefore provide a valuable treatment strategy for IGF1R inhibitor-based therapies for IGF1 signaling-associated tumors [15].

The existence of cell-free nucleic acids (including cell-free DNA/cfDNA/sequences) in human blood, as well as in urine, saliva, or feces, is a known fact [16]. The methylation status or fragmentation of cfDNAs may carry information about their source [17]. In terms of their origin, cfDNAs fall into several categories. Endogenous cfDNA sequences are derived from tissues and cells, whereas exogenous sequences are primarily derived from the host microbiome, infectious agents, the fetus, and food [18–20]. Toll-like receptors (TLRs) are innate immune receptors [21]. TLR9 is capable of detecting DNA from both endogenous and exogenous sources [21]. We have previously demonstrated that the structural modifications of self-DNA (e.g., methylation status and fragment length) play a significant role in activation of the TLR9-mediated signaling pathways [22]. However, little data is available on the relationship between Toll-like receptor signaling and the IGF1R-associated pathway. It was demonstrated that exposure to CpG-oligodeoxynucleotide (CpG-ODN), a ligand of TLR9, can increase the expression of IGF1 in intestinal epithelial cells [23]. IGF1 further contributes to the intestinal homeostasis by inducing macrophages with immune suppressive properties [23]. These data are in line with the finding that the physiologic TLR9/CpG-ODN-DNA interaction is essential for the homeostasis of the intestinal immune system [24].

In HT29 colon cancer cells, a close interplay between self-DNA-induced TLR9 signaling and autophagy response was found, with notable effects on cell survival and differentiation [25]. However, the interrelated role of IGF1R inhibition and TLR9/autophagy signaling in HT29 colon cancer cells has not yet been clarified. Therefore, we aimed to assess this complex interaction in HT29 cells.

We decided to use HT29 cells because of several aspects. There is basal TLR9 expression in HT29 cells, which is essential for induction with self-DNA [23]. Since DNA treatment via TLR9 can exert its effects in both MyD88-dependent and MyD88-independent ways, it is also important that the MyD88-dependent and MyD88-independent TLR signaling pathways are intact in HT29 cells [26]. IGF1R expression in HT29 cells is moderate as compared to other CRC cell lines (e.g., SW480 or DLD-1) [27]. Also, in HT29 cells, elevated IGF2 expression can be detected, which is essential for both autocrine activation of IGF1R signaling and studying the effect of IGF1R inhibition [28]. The HT29 cells adequately represent sporadic colon cancers [29]. Not all colorectal cancer cell lines meet these criteria.

Here, we found that the concomitant use of tumorous self-DNA and IGF1R inhibitor displays anti-proliferative potential, which can be inhibited by parallel TLR9 signaling inhibition. The different effects of IGF1R, TLR9, and autophagy inhibitors alone or in combination on HT29 cell proliferation and autophagy suggest that the IGF1R-associated and non-IGF1R-associated autophagy machinery is “Janus-faced” regarding its effect on cell proliferation. Autophagy induced by different combinations of self-DNA and inhibitors may result in the survival of some CD133-positive stem-like HT29 cancer cells, which may play an important future role in the recurrence of CRC.

## Materials and Methods

### Maintenance of HT29 Cell Culture and Self-DNA Isolation

The HT29 undifferentiated colon adenocarcinoma cell line was purchased from the 1st Department of Pathology and Experimental Cancer Research (Semmelweis University, Budapest, Hungary). The cells were maintained in RPMI 1640 medium (Sigma-Aldrich, United States) supplemented with 10% (v/v) fetal bovine serum (FBS; Standard Quality; PAA Laboratories GmbH, Austria), 125 μg/ml amphotericin B (Sigma-Aldrich, United States), and 160 μg/ml gentamycin (Sandoz, Sandoz GmbH, Austria). The medium was replaced every second day.

Genomic DNA (gDNA; g) was isolated from 5 × 10^7^ steady state, proliferating HT29 cells. DNA isolation was performed by using a High Pure PCR template preparation kit containing proteinase K (Roche GmbH, Germany). The DNA samples were treated with 5 μl RNase A/T1 Mix (Thermo Scientific, Germany). The DNA concentration was determined by Nanodrop (Thermo Scientific, Germany). Gel electrophoresis determined that the fragment length of gDNA was approximately 10,000 base pairs [22].

According to the bisulfite sequencing analysis of Ogoshi et al. [30], the basal methylation status of HT29 cells’ CpG sites is as follows: 31.6% in the low range; 11.6 percent in the middle; and 56.7 percent in the high range. Based on MALDI-TOF mass spectrometry measurements, the DNA samples were free of RNA, protein, or lipopolysaccharide contamination (data not shown).

### HT29 Cell Treatments

For incubation with the DNA samples, 0.5 × 10^6^ HT29 cells were placed into a 12-well-plate in RPMI 1640, supplemented with amphotericin B, gentamycin, and FBS, as described above. After 24 h, the starting medium was changed to RPMI 1640 with gentamycin but without FBS. Aliquots of 15 μg of self-DNA were separately dissolved in 200 μl sterile phosphate buffered saline (PBS).

HT29 cells were incubated with the self-DNA samples at 37°C in a humidified atmosphere of 5% CO_2_ and 95% O_2_. For the control cells, only 200 μl sterile PBS was added. After 72 h, cells were washed twice with 5 ml of sterile PBS and resuspended in a final volume of 5 ml of PBS.

#### Inhibition of TLR9 and IGF1R Signaling

To inhibit TLR9 or IGF1R signaling, HT29 cells were pretreated for 1 h with TLR9 antagonist (O) (5 μM ODN2088; Invivogen, CA, United States) or picropodophyllin (P) (0.05 μM; BML-EI372-0001; EnzoLifeSciences, BioMarker Kft., Gödöllő, Hungary; diluted in dimethyl sulfoxide/DMSO; Sigma-Aldrich Budapest, Hungary/) for 1 h before treatments with DNA. All treatments were performed in triplicate. Between plates, two samples received the same treatment to avoid possible manual errors in the treatments between plates.

#### Inhibition of Autophagy

Chloroquine (C), an anti-inflammatory substance, is the most commonly used autophagy completion inhibitory drug to ascertain autophagic flux because of its suitability *in vivo* [31]. HT29 cells were treated with chloroquine (10 μM; C6628 Sigma-Aldrich, Budapest, Hungary; diluted in DMSO) for 1 h before treatments with DNA. All treatments were performed in triplicate. Between plates, two samples received the same treatment to avoid possible manual errors in the treatments between plates.

The treatment plan for HT29 cells is shown in Table 1.

**TABLE 1:** Treatment plan for HT29 cancer cells. gDNA, genomic deoxyribonucleic acid; ODN, O, CpG oligonucleotide; K, non-treated, control; P, picropodophyllin; C, chloroquine; Kg, gDNA control; gO, gDNA + ODN2088; gP, gDNA + picropodophyllin; gC, gDNA + chloroquine; gOP, gDNA + ODN2088 + picropodophyllin; gPC, gDNA + picropodophyllin + chloroquine.

Sample groups	GDNA	ODN2088	Picropodophyllin	Chloroquine
K	**−**	**−**	**−**	**−**
O	**−**	**+**	**−**	**−**
P	**−**	**−**	**+**	**-**
C	**−**	**−**	**−**	**+**
Kg	**+**	**−**	**−**	**−**
gO	**+**	**+**	**-**	**−**
gP	**+**	**−**	**+**	**−**
gC	**+**	**−**	**−**	**+**
gOP	**+**	**+**	**+**	**−**
gPC	**+**	**−**	**+**	**+**

### Measuring Cell Viability and Proliferation

The use of the Alamar Blue assay served a dual purpose: partly to examine cell viability (metabolic activity) and partly to study cell proliferation [32].

The anti-proliferative effects of the treatments were measured after a 4 h incubation period using Alamar Blue (Thermo Fisher Scientific, Budapest, Hungary). The fluorescence was measured at 570–590 nm (Fluoroskan Ascent FL fluorometer; Labsystems International Ltd., Budapest, Hungary) and the results were analyzed by the Ascent Software.

As metabolic activity is not necessarily proportional to proliferative activity, direct cell counts (average cell numbers determined by using Bürker-Türk counting chambers) were also performed in the examined cell groups to determine the proliferative activity compared to the control sample. We used Trypan Blue dye (302643 Sigma-Aldrich, Budapest, Hungary) to exclude dead cells.

### Semithin Sections

From the HT29 cell blocks fixed for TEM semithin sections were cut for viewing by a digital microscope. The sections were stained with toluidine blue (toluidine blue O 4 g, pyronin 1 g, borax 5 g in distilled water). Semithin sections were then digitalized using a high-resolution PANNORAMIC 1000 FLASH DX instrument (3DHISTECH Ltd., Budapest, Hungary), and analyzed with CaseViewer software (3DHISTECH Ltd., Budapest, Hungary). The average number of proliferative cells was counted in five fields of view per sample (mean ± SD/sample).

### Total mRNA Isolation and NanoString Analysis

Total mRNA from HT29 cells was extracted with the RNeasy Mini Kit (Qiagen, United States) according to the manufacturer’s instructions. Quantitative (Nanodrop) and qualitative analysis (Bioanalyzer Pico 600 chip kit RNA program; RIN > 8 in all cases) were performed.

mRNA samples required for gene expression assays of HT29 cells were prepared by tripling the treated groups. In HT29 samples, cell numbers ranged from 100,000 to 11,135,000 per well, and the recovered mRNA concentration ranged from 8 to 256 ng/μl/sample. mRNAs recovered from triplicates were pooled and used in the NanoString assay.

The custom mRNA Assay Evaluation panel (NA-SPRINT-CAR-1.0, nCounter SPRINT Cartridge) containing our custom gene code set (NA-XT-GXA-P1CS-04 nCounter GX Custom CodeSet) was designed by NanoString (the order was placed through Biomedica Hungaria Ltd., Budapest, Hungary). The NanoString experiments were carried out by RT-Europe Research Center Ltd. (Mosonmagyaróvár, Hungary; website: http://rt-europe.org/) as part of a contract work.

In brief, as input material, a total of 50 ng (10 ng/µl) RNA samples were denatured at 85°C for 5 min and cooled on ice. 5 µl samples were added to 8 µl mix containing the Reporter code set in Hybridization buffer. 2 µl Capture ProbeSet was added to each tube and incubated at 65°C for 18 h. The resulting reaction vials were supplemented with 17 µl molecular biology grade dH2O. 30 µl of the resulting sample mixes were loaded into the microfluidic sample chambers of the nCounter SPRINT Cartridge (SPRINT-CAR-1.0, Nanostring) directly before initiating the run on the nCounter Sprint Profiler. Primary data analysis was performed using the nSolver 4.0 Analysis software.

The criterion for selecting the genes to be examined was to establish an association between IGF1R and TLR9 signaling, apoptosis, cell proliferation, autophagy, and cancer cell stemness.

#### The Gene Set Contained the Following Genes (With Probe NSIDs)

IGF1R signaling pathway: IGF1R (Insulin-like growth factor 1 receptor; NM_000875); MAPK (Mitogen-activated protein kinase; NM_002755.2:970); PI3K (Phosphoinositide 3-kinase; NM_006218.2:2445); Akt (Ak strain transforming; NM_001014432.1:1275).

TLR9 signaling pathway: TLR9 (Toll-like receptor 9; NM_017442); MyD88 (Myeloid differentiation factor 88; NM_002468), NF-kB (nuclear factor-kappa B; NM_003998).

Extrinsic and intrinsic apoptosis-related genes: Caspase-3 (NM_004346.3:2156), Caspase-8 (NM_033355), Caspase-9 (NM_032996).

Anti-apoptotic and autophagy suppressor genes: PI3K (Phosphoinositide 3-kinase; NM_006218.2:2445), Akt (Ak strain transforming; NM_001014432.1:1275), mTOR (Mechanistic/mammalian target of rapamycin; NM_004958.3:1865), Bcl-2 (B-cell lymphoma 2; NM_000657.2:5).

Pro-apoptotic and autophagy activator genes: MAPK (Mitogen-activated protein kinase; NM_002755.2:970), AMPK (AMP-activated protein kinase; NM_006251.5:366), Bax (BCL2 associated X; NM_138761.3:342).

Autophagy genes: Beclin1 (NM_003766.2:810), ATG16L1 (Autophagy related 16 like 1; NM_017974.3:2405), MAP1LC3B (Microtubule-associated proteins 1A/1B light chain 3B; NM_022818.4:1685), ULK1 (Unc-51 like autophagy activating kinase; NM_003565.1:465), Ambra-1 (activating molecule in beclin-1-regulated autophagy; NM_017749).

HT29 cancer cell stemness-related gene: CD133 (NM_006017).

Housekeeping genes: C1orf43 (NM_015449.2:477), CHMP2A (NM_014453.3:241), PSMB2 (NM_002794.3:639), RAB7A (NM_004637.5:277), REEP5 (NM_005669.4:280), SNRPD3 (NM_004175.3:309), VCP (NM_007126.2:615), VPS29 (NM_016226.4:565).

### Taqman Real-Time Polymerase Chain Reaction Analysis

For validating the NanoString gene expression analysis method, mTOR (ID: Hs00234508_m1), ATG16L1 (ID: Hs01003142_m1), LC3B (ID: Hs00797944_s1), BCN1 (ID: Hs01007018_m1), IGF1R (ID: Hs00609566_m1) and TLR9 (ID: Hs00370913_s1) triplicated Taqman real-time polymerase chain reactions were used in an Applied Biosystems Micro Fluidic Card System. The measurements were performed using an ABI PRISM 7900HT Sequence Detection System as described in the product’s User Guide (http://www.appliedbiosystems.com, CA, United States). Gene expression levels for each individual sample were normalized to GAPDH (ID: Hs02786624_g1) expression. Mean relative gene expression was determined and differences were calculated using the 2−ΔC(t) method. The whole cycle number was 45.

### IGF1R, CD133, and Autophagy Immunocytochemistry

To detect IGF1R, CD133, and autophagy-associated ATG16L2, Beclin1, and LC3 protein expression, HT29 cell smears were incubated for 1 h at 37°C with optimally diluted anti-IGF1R monoclonal antibody (Chemicon International; Clone: 24-31; 1:50 dilution in PBS), anti-CD133/1-biotin antibody (1:100, Miltenyi), and anti-ATG16L1-, anti-BECN1-, and anti-MAP1LC3B antibodies (1:200, Antibody Verify, LA, USA). After rinsing 3 times with PBS, cell smears were finally treated with an anti-rabbit EnVision polymerHRP conjugate kit (K4003, DAKO) for 40 min. Secondary immunodetection was performed with EnVision System Labelled Polymer–HRP K4001 (Anti-Mouse 1/1; DAKO), as described in the manual. Signal conversion was carried out with the Liquid DAB + Substrate Chromogen System (DAKO). After rinsing in PBS, hematoxylin co-staining was performed. Cell smears were then digitalized using a high-resolution PANNORAMIC 1000 FLASH DX instrument (3DHISTECH Ltd., Budapest, Hungary), and analyzed with CaseViewer software (3DHISTECH Ltd., Budapest, Hungary). The immunocytochemistry analyses were carried out as part of contract work (Pathology Laboratory, Heim Pál National Institute of Pediatrics, Budapest, Hungary).

### WES Simple Western Blot

WES Simple (ProteinSimple 004-600, Minneapolis, MN, USA) analysis was also performed. A 12–230 kDa Separation Module (ProteinSimple SM-W004) was used for all the proteins (Phospho-mTOR (Ser2448) Rabbit Antibody/Cell Signaling; #2971; 1:50 dilution; 199 kDa/; mTOR (7C10) Rabbit mAb/Cell Signaling; #2983; 1:50 dilution; 200 kDa/; Atg16L1 (D6D5) Rabbit mAb/Cell Signaling; #8089; 1:50 dilution; 66.68 kDa/; Beclin-1 (D40C5) Rabbit mAb/Cell Signaling; #3495; 1:50 dilution; 60 kDa/; LC3B (D11) XP Rabbit mAb/CellSignaling; #3868; 1:50 dilution; 14.16 kDa/; Anti-β-Actin (AC-74) Mouse mAb/SigmaAldrich; A2228; 1:50 dilution; 48 kDa/), and either the Anti-Rabbit Detection Kit (ProteinSimple DM-001) or Anti-Mouse Detection Kit (ProteinSimple DM-002) were used, depending on the primary antibodies. Briefly, based on the used primary antibodies, 0.2 or 1 μg/μl cell lysates were diluted in 0.1× WES Sample Buffer (ProteinSimple 042-195), and Fluorescent Master Mix (1:4, ProteinSimple PS-FL01-8) was also added. Following a 5-min incubation at 95°C, the Antibody Diluent (ProteinSimple 042-203), primary and secondary antibodies, and chemiluminescent substrate were applied to the WES capillary plate. The WES system settings were (a) stacking and separation (395 V, 30 min), (b) blocking (5 min), (c) incubations with primary and secondary antibodies (30 min) and (d) luminol/peroxide chemiluminescence detection (15 min) (the exposure time was 2 s). The electropherograms were manually corrected if required for the evaluations.

### Cell Counting and Interpretation of Immunoreactions

At ×200 magnification, 10 fields of view and at least 100 cells (mainly 110 cells) per field of view were examined in a semiquantitative manner in each digitalized sample. The percentage of immunopositivity and non-immunoreactive HT29 cells was determined.

In the case of the IGF1R immune response, weak, moderate, and strong membrane staining and perinuclear cytoplasm staining were examined. As for autophagy, weak, moderate, and strong ATG16L1 and Beclin1 homogenous or spotted immunoreactions were detected in the cytoplasm. In the case of LC3, weak, moderate, and strong punctuated or spotted cytoplasmic immunoreactions were observed. The notation “−/+” indicates non-immunoreactive and weakly immunopositive cells. The designation “++/+++” indicates moderately or strongly immunopositive cells.

### Transmission Electron Microscopy for Evaluation of Autophagy

HT29 cells in the wells were fixed in 2% glutaraldehyde (0.1 M Millonig buffer, pH 7.4) for 60 min. After washing with both 0.1 M of phosphate buffer (3 times for 5 min) and 0.1 M pH 7.2 sodium cacodylate buffer (3 times for 5 min), the samples were post-fixed with 1% osmium tetroxide in 0.1 M sodium-cacodylate buffer for 60 min at 4°C in the dark. After washing 3 times for 5 min with sodium-cacodylate buffer (pH 7.4), the cells were pelleted by centrifugation and embedded in 10% gelatin in phosphate buffer (pH 7.4). After dehydration in a graded series of alcohol, the samples were embedded into Poly/Bed epoxy resin. Ultrathin sections (70–80 nm) were contrast stained with uranyl acetate and lead citrate, respectively. Ultrastructural analyses were performed by using a JEM-1200EXII Transmission Electron Microscope (JEOL, Akishima, Tokyo, Japan).

The average number of autophagic vacuoles was counted in five HT29 cells per sample (mean ± SD/cell).

### Statistical Analysis

At least three independent experiments were conducted. Data of cell viability, cell number, and proliferation were presented as means ± SD. The Chi2-test and Student’s t-test were used for statistical analyses. *p* < 0.05 was considered statistically significant. In the case of immunocytochemistry, statistical analysis with one-way ANOVA and Tukey HSD test was performed by using R Core Team [33].

Regarding NanoString gene expression analysis, after importing RCC files to the nSolver Analysis Software, quality checking was performed. Then agglomerative cluster heat maps were created. The Euclidean distance metric was used to calculate the distance between two samples (or genes) as the square root of the sum of squared differences in their log count values. The average linkage method was used to calculate the distance between two clusters.

## Results

### Cell Viability and Proliferation Measurements

The metabolic activity of the HT29 cells was significantly (*p* < 0.05) increased in all treatment groups except the gOP combination as compared to K (control, non-treated cells). The highest metabolic activity was found for P treatment.

The significantly lowest (*p* < 0.05) cell proliferation was found in the Kg treatment group of cells, as compared to K.

When gDNA, ODN2088, picropodophyllin, and chloroquine treatments were co-administered (i.e., gO, gP, gC), effective inhibition of HT29 cell proliferation with high metabolic activity was observed. The combination of gDNA, ODN2088, and picropodophyllin (i.e., gOP) raised proliferative activity back to levels close to non-treated control group.

**FIGURE 1:**
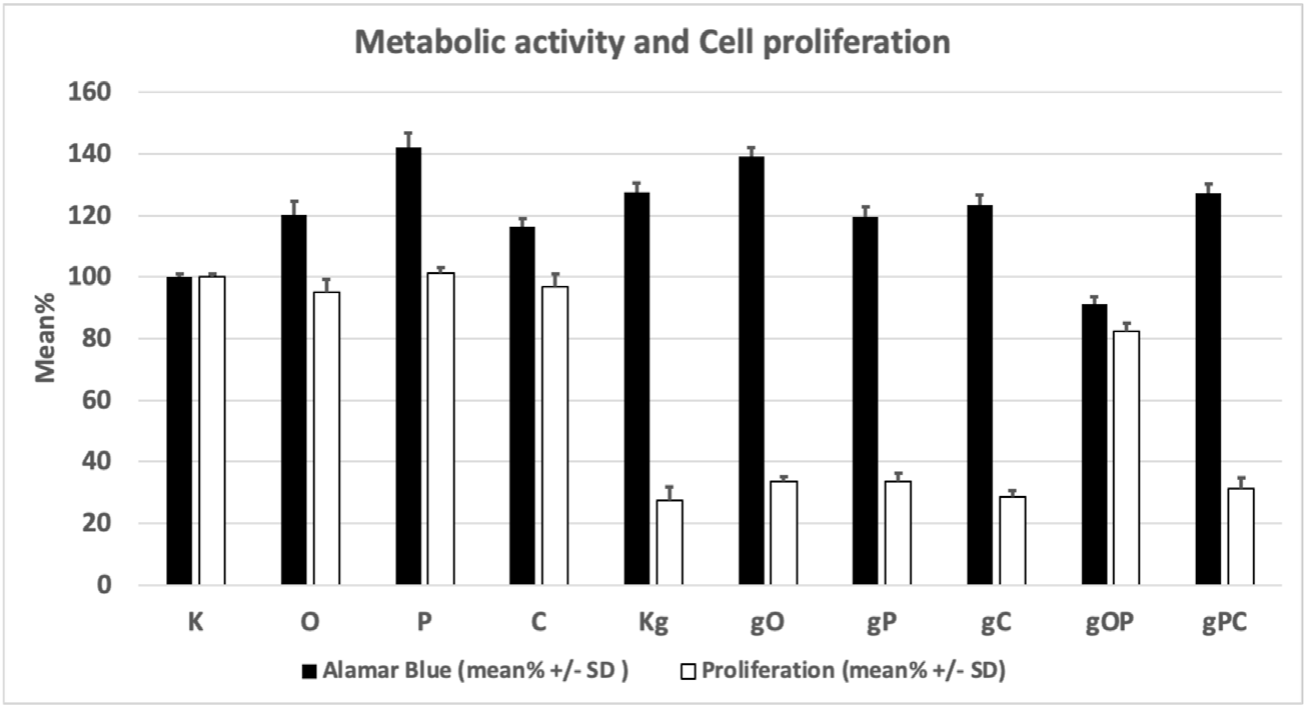
Changes in the metabolic activity and proliferation of the studied cell groups under the influence of each treatment combination. K, control, non-treated HT29 cells; g, genomic deoxyribonucleic acid; Kg, gDNA-treated HT29 cells; O, ODN2088 CpG oligonucleotide; P, picropodophyllin; C, chloroquine; SD, standard deviation.

Viability, cell number, and proliferation data are illustrated in Table 2 and Figure 1.

**TABLE 2:** Numerical data of viability, cell number, and proliferation.

Sample	Alamar blue (mean% ± SD)	Average cell number/350 μl (±SD)	Proliferation% (±SD)
K	100 ± 1.1	800000 ± 8800	100 ± 1.1
O	120.17 ± 4.5^a^	760000 ± 32680	95 ± 4.3
P	142.15 ± 4.7^a^	810000 ± 25920	101.25 ± 1.8
C	116.23 ± 2.9^a^	775000 ± 31775	96.87 ± 4.1
Kg	127.51 ± 3.1^a^	220000 ± 9900	27.5 ± 4.5*
gO	139 ± 3.1^a^	270000 ± 3780	33.75 ± 1.4*
gP	119.57 ± 3.2^a^	270000 ± 7020	33.75 ± 2.6*
gC	123.55 ± 3.1^a^	230000 ± 4370	28.75 ± 1.9*
gOP	91.3 ± 2.4^a^	660000 ± 16500	82.5 ± 2.5*
gPC	127.38 ± 2.8^a^	250000 ± 9250	31.25 ± 3.7*

a Represents significant alteration as compared to K (control, non-treated sample) (*p* < 0.05).

g, genomic deoxyribonucleic acid; O, ODN2088 CpG oligonucleotide; P, picropodophyllin; C, chloroquine; SD, standard deviation.

### Semithin Sections

To investigate whether the changes in cell numbers after treatments with genomic DNA and/or TLR9, IGF1R, or autophagy inhibitors were due to low proliferation activity or increased cell death, semi-thin sections were also examined in selected cases.

**FIGURE 2:**
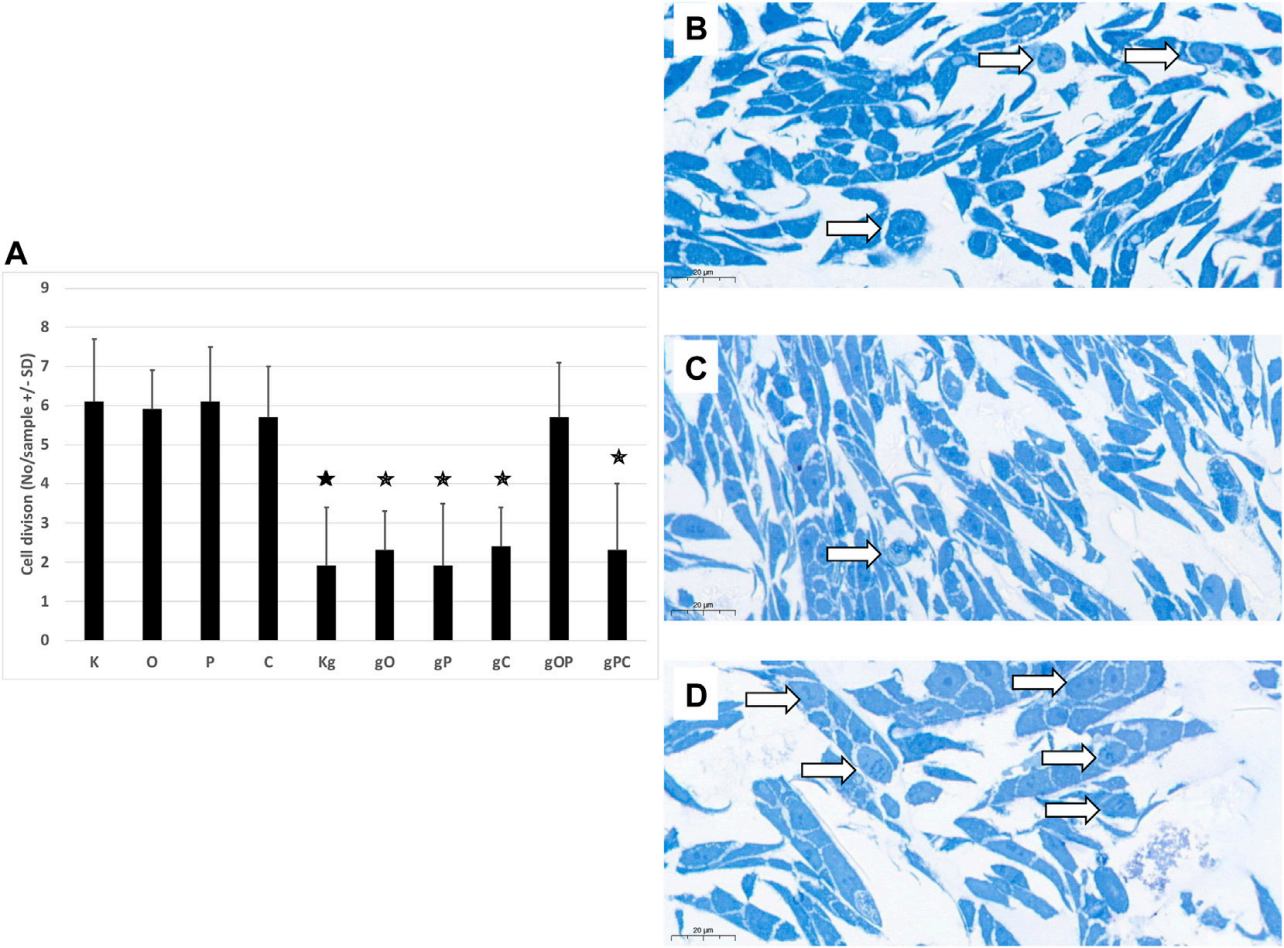
Cell divisions in HT29 cells. **(A)** The graph indicates the average number of cell divisions per sample in the different treatment groups. The representative image inserts highlight the signs of cell division in HT29 cells. **(B)** Control, non-treated cells; **(C)** gDNA-treated control cells; **(D)**: larger number of cell divisions in gOP sample. K, control, non-treated HT29 cells; O, ODN2088; P, picropodophyllin; C, chloroquine; Kg, genomic DNA control; g, genomic DNA; arrows indicate cell divisions; scale bar represents 20 μm; the presence of stars indicates significant differences from K (*p* < 0.05).

In the case of incubation with gDNA, the incidence of proliferation was proportional to the cell numbers obtained. When gDNA was co-administered with picropodophyllin and/or chloroquine, remarkably reduced proliferative activity was observed. In contrast, after combining gDNA with ODN2088 and picropodophyllin, higher proliferative activity was detected **(** Figures 2A–D).

### NanoString and Taqman Gene Expression Analyses

Regarding TLR9 mRNA expression, gDNA treatment resulted in TLR9 upregulation as compared to untreated control cells. As for IGF1R gene expression, gDNA treatment did not increase the expression as compared to untreated control. Otherwise, incubation with gDNA displayed a similar gene expression profile as seen in control samples.

Concerning the effect of combined IGF1R inhibition and gDNA treatment on the expression of IGF1R signaling elements, slight IGF1R overexpression was found without any substantive difference in MAPK, PI3K, and Akt expressions. Except for TLR9 in gC and Bcl2 in gO, gDNA combined with ODN2088 or chloroquine resulted in remarkable overexpression of all examined genes.

Because cell-free DNA treatment affects both TLR9 signaling and the autophagy machinery, we also examined how the effect of concomitant IGF1R inhibition and gDNA treatment is altered by the inhibition of TLR9 signaling or autophagy.

gDNA in combination with picropodophyllin and chloroquine caused the most pronounced increase in TLR9 signaling related (i.e., MyD88, NF-kB), autophagy-related (i.e., ATG16L1, Beclin1, MAP1LC3B, ULK1, Ambra-1), autophagy suppressor/anti-apoptotic (i.e., PI3K, Akt, mTOR), and autophagy activator/pro-apoptotic (i.e., MAPK, AMPK, Bax) gene expressions, except that of TLR9 and Bcl2. On the contrary, gDNA combined with ODN2088 and picropodophyllin resulted in general down-regulation of the assayed genes, with the exception of TLR9 and Bcl2.

**FIGURE 3:**
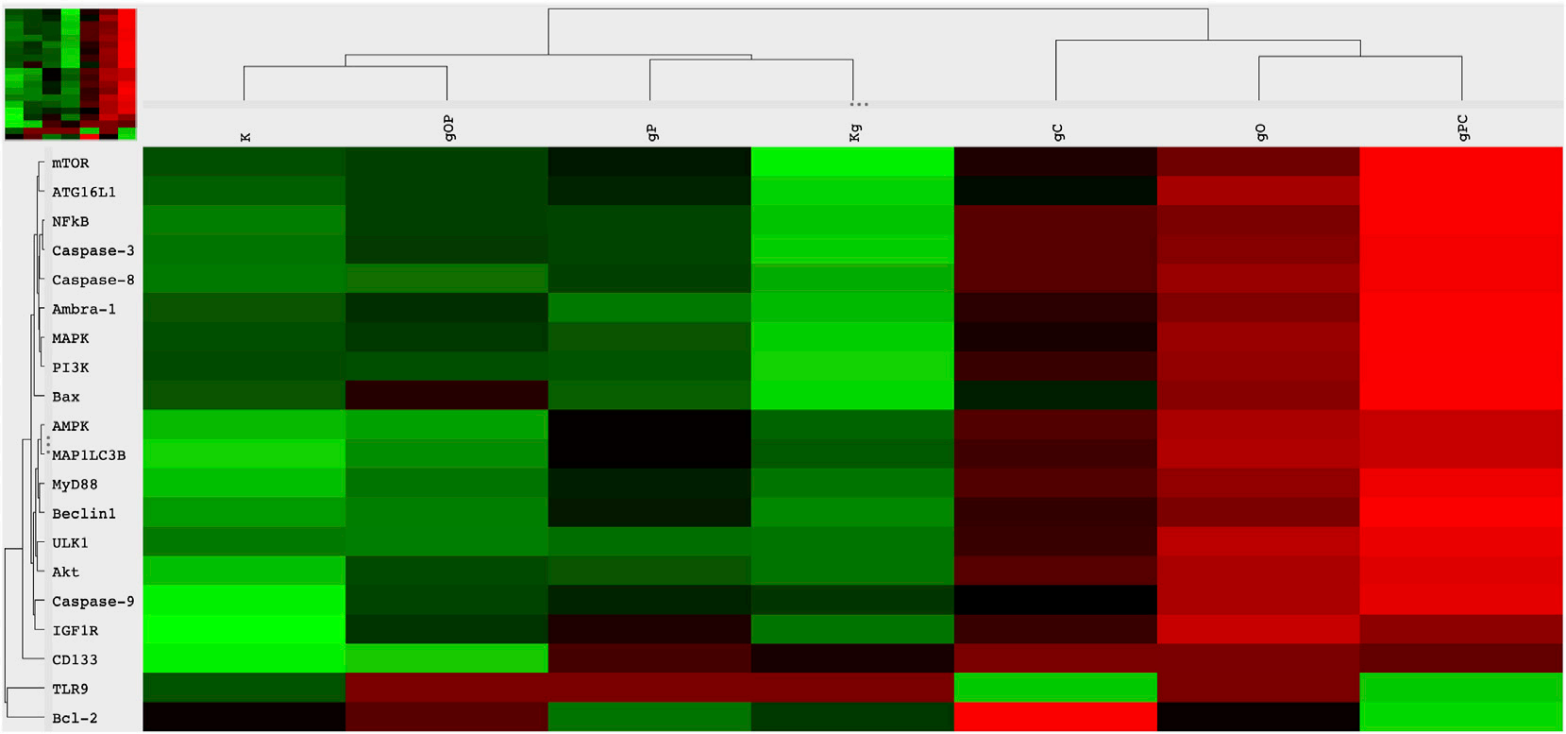
Heatmap visualization of the NanoString gene expression analysis. Gene expression alterations in HT29 cells after incubation with genomic self-DNA. g, genomic deoxyribonucleic acid (gDNA); O, ODN2088—TLR9 inhibitor; P, picropodophyllin; C, chloroquine; K, control, non-treated HT29 cells; Kg, gDNA treated control HT29 cells; red, overexpression, green, downregulation.

The cancer stemness-related gene, CD133, was overexpressed in the case of gP, gC, gO, and gPC treatment combinations. Alterations in gene expression are visualized in Figure 3.

**FIGURE 4:**
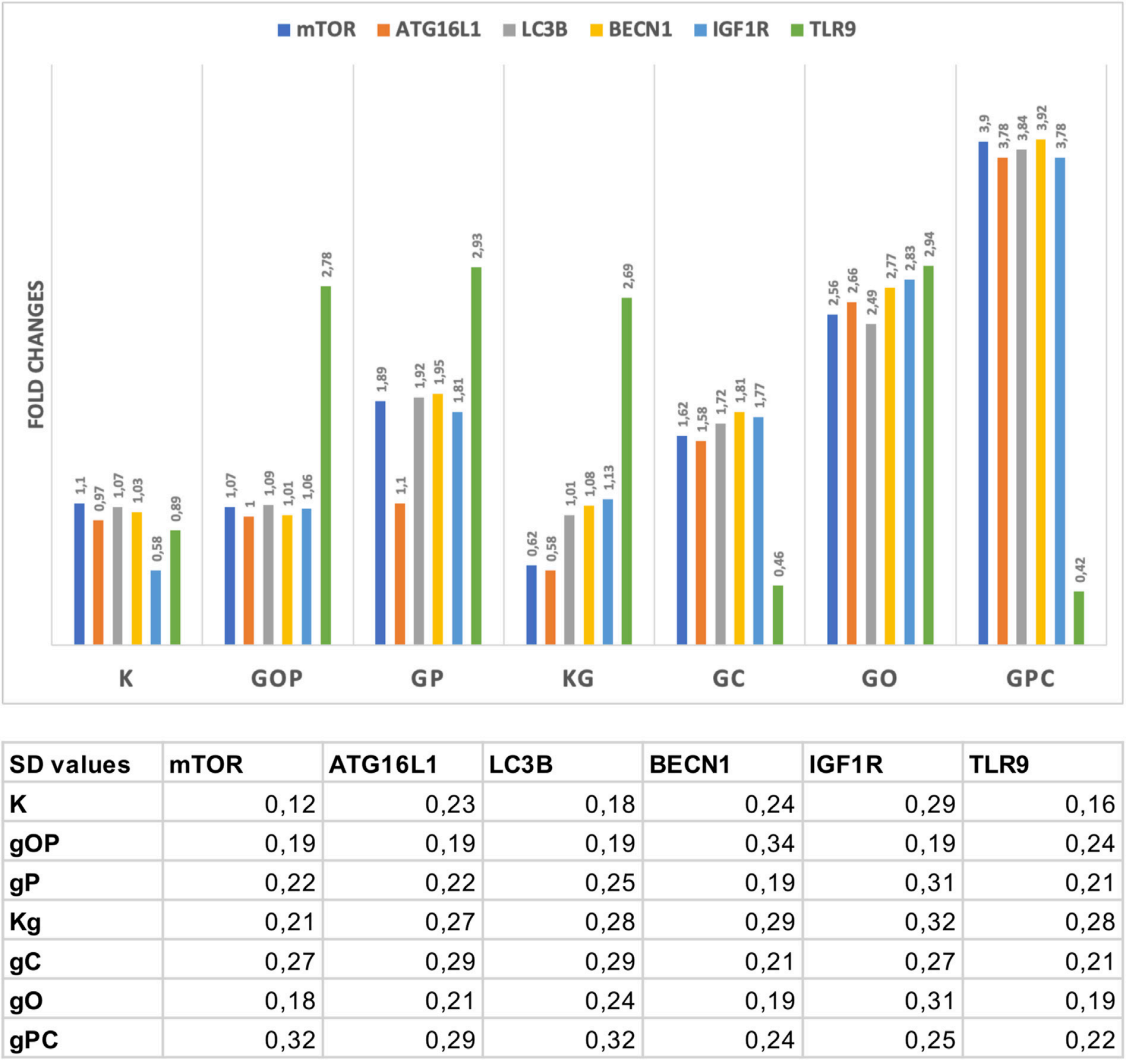
Fold changes in the assayed genes. The gene expression fold changes were in correlation with the NanoString gene expression results. The table indicates the SD values in each case. G/g: genomic deoxyribonucleic acid (gDNA); O, ODN2088—TLR9 inhibitor; P, picropodophyllin; C, chloroquine; K, control, non-treated HT29 cells; Kg, gDNA treated control HT29 cells.

The results of Taqman RT-PCR validated the gene expression alterations detected by NanoString/nCounter analysis. Fold changes of the analyzed gene expressions are summarized in Figure 4.

### Immunocytochemistry Analysis and WES Simple Western Blot

Regarding IGF1R and autophagy-related genes (i.e., ATG16L1, Beclin1, and MAP1LC3B), we performed immunocytochemistry to validate gene expression results at the protein level.

First, we observed that the distribution of non-immunoreactive and weak immunopositive (“−/+”), as well as moderately and strongly immunoreactive (“++/+++”) HT29 cells was in correlation with the gene expression results. In the case of IGF1R, moderate to strong immunopositivity was detected to a greater extent after incubation with gO, gC, and gPC. As for autophagy, strong upregulation of ATG16L1 protein expression was found in the gO and pPC groups, followed by the Kg, gP, and gOP treatments. The highest proportion of strong Beclin1 and LC3 immunoreactivity was detected in the gO and gPC groups, followed by the gP and gC treatments.

**FIGURE 5:**
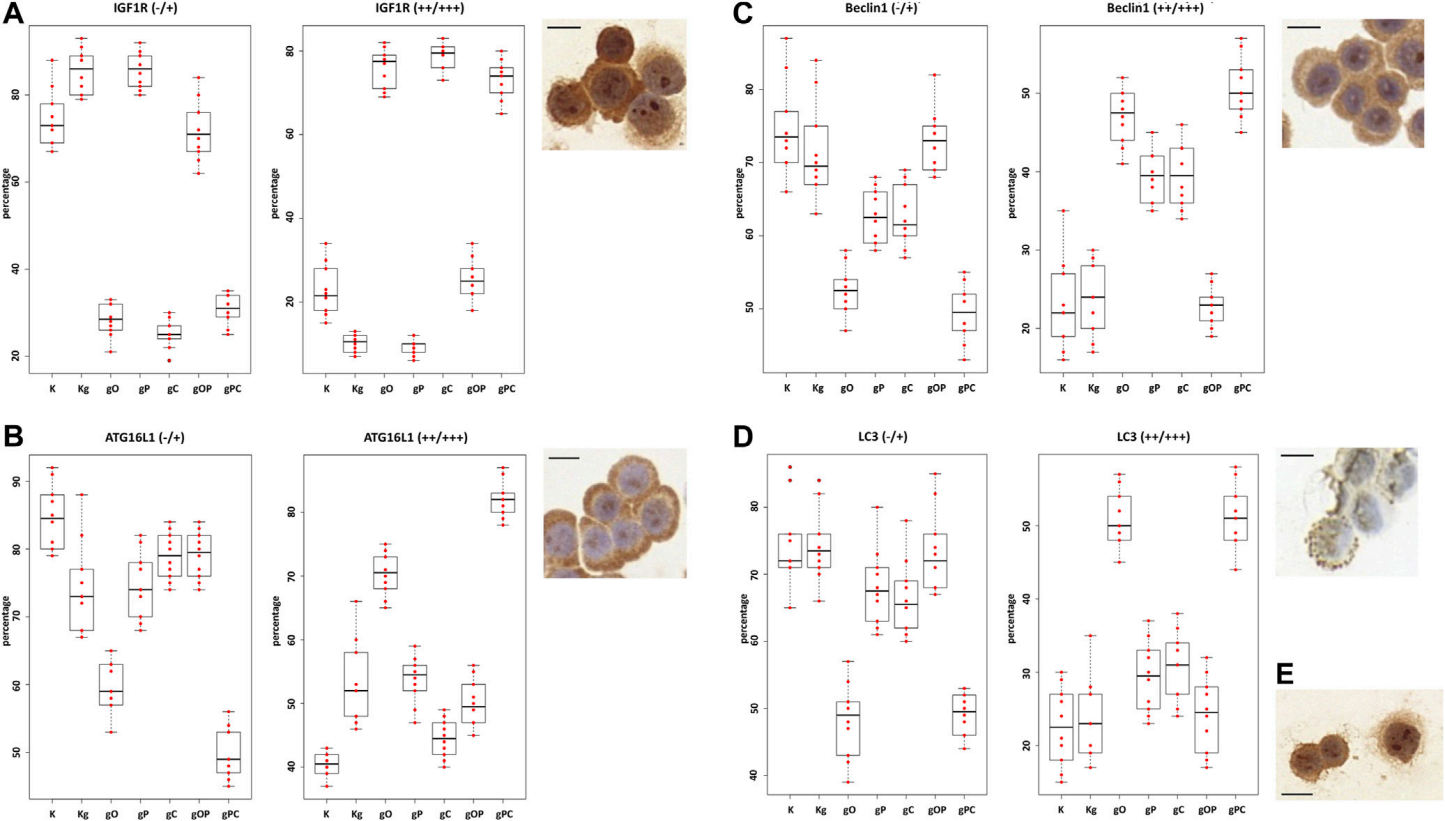
One-way ANOVA results of IGF1R, ATG16L1, Beclin1, and LC3 immunocytochemistry analyses. The percentages of non-immunoreactive and weakly immunopositive (“−/+”), as well as moderately and strongly immunopositive (“++/+++“) HT29 cells within the treatment groups were plotted on box and whisker plots. **(A)** The boxplots for IGF1R. The right upper inserts represent the moderate to strong IGF1R immunopositivity (at ×200 magnification; the scale bar indicates 10 μm). The boxplots and representative immunostainings for ATG16L1 **(B)**, Beclin1 **(C)**, and LC3B **(D)** are also visualized. **(E)** Right lower insert represents CD133 positive HT29 cells (×200 magnification; the scale bar indicates 10 μm). K, control, non-treated HT29 cells; Kg, genomic DNA (gDNA) treated control; gO, gDNA + ODN2088; gP, gDNA + picropodophyllin; gC, gDNA + chloroquine; gOP, gDNA + ODN2088 + picropodophyllin; gPC, gDNA + picropodophyllin + chloroquine.

Based on the NanoString gene expression results, we examined whether there was an HT29 cell expressing CD133 protein in each treatment group. CD133-positive cells were found only scattered in the gO, gP, gC, and gPC treatment groups. The results of the one-way ANOVA test and the representative immunocytochemistry images are visualized in Figure 5. The results of the Tukey HSD test can be seen in Supplementary Figure S1.

**FIGURE 6:**
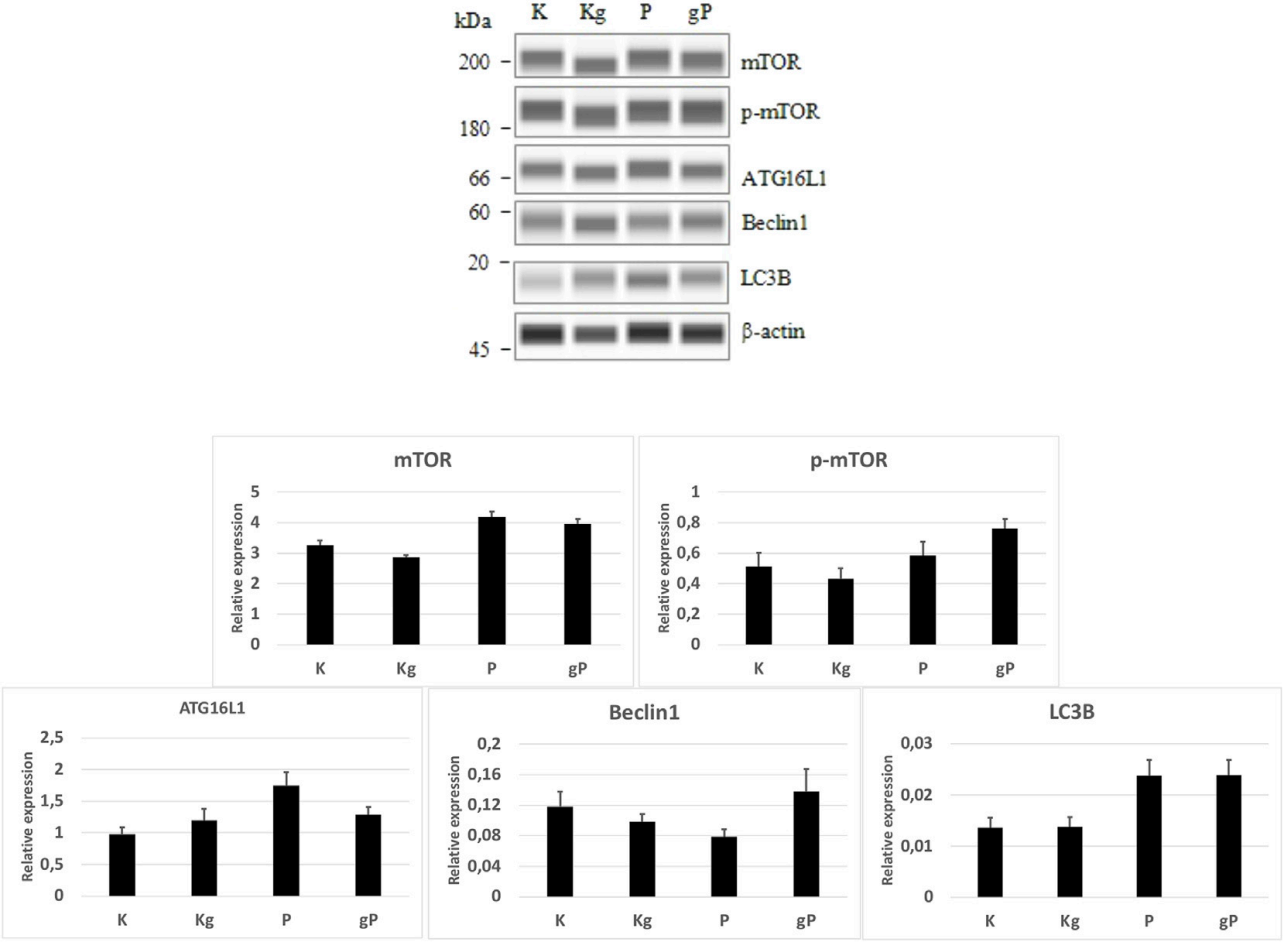
WES Simple Western blot analyses of selected proteins. According to protein expression values normalized to β-actin (bar graphs), the mTOR and phospho-mTOR (p-mTOR) protein activities, as well as the autophagy-related protein (ATG16L1, Beclin1, LC3B) expressions were in relation to the gene expression results. K, control, non-treated HT29 cells; P, picropodophyllin; Kg, genomic DNA control; g, genomic DNA.

In our experiment, by adding picropodophyllin to gDNA-treated cells, the relatively smaller decrease in PI3K, Akt, AMPK, and mTOR gene expressions resulted in a potential inhibitory effect on autophagy initiation. If autophagy is inhibited, mTOR must be active, so a WES Simple Western blot was also performed. In the cases of K, Kg, and gP groups, the mTOR, phospho-mTOR, and autophagy-related protein expressions, together with phospho-mTOR activity, were in relation to the gene expression results (Figure 6).

### Transmission Electron Microscopy

Control, non-treated, metabolically active HT29 cells (3 ± 1 pieces/cell), similarly to chloroquine-treated controls (4 ± 1.5 pieces/cell), displayed autophagic vacuoles (AVs) in the cytoplasm, indicating macroautophagy. The frequency of AVs in ODN2088 (7 ± 1.4 pieces/cell) control cells was higher as compared to control. However, in picropodophyllin-treated control cells AVs were only scattered (0.5 ± 0.5 piece/cell). Incubation with gDNA resulted in the appearance of a more intense macroautophagy (6 ± 2 pieces/cell), and co-administration of ODN2088 (10 ± 2.2 pieces/cell) or chloroquine (5 ± 1.5 pieces/cell) also favored the presence of intense autophagy, represented by disorganized cell structure along with chromatin condensation and blebbing. The combination of gDNA with picpropodophyllin and/or ODN2088, similarly to non-treated control cells, resulted in low number of AVs (2 ± 1.5 pieces/cell in gP; 3 ± 1 pieces/cell in gOP). On the contrary, gDNA, picropodophyllin and chloroquine co-administration caused an intense macroautophagy (11 ± 2.6 pieces/cell).

In the case of the gDNA + picropodophyllin combination, multivesicular bodies (MVBs) were also detected.

**FIGURE 7:**
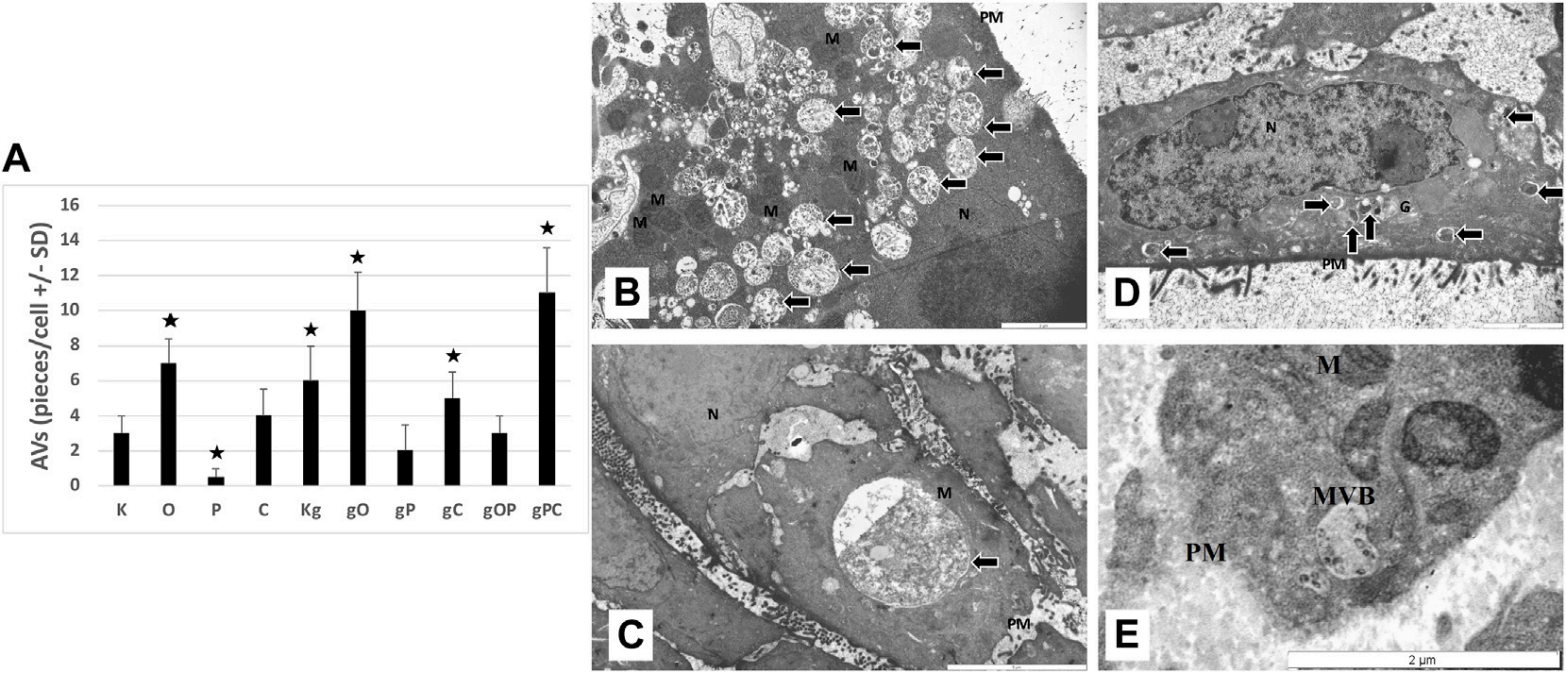
Transmission electron microscopy results. **(A)** The graph indicates the average number of autophagic vacuoles (AVs; pieces/cell) in the different treatment groups. The representative image inserts highlight the autophagy-related structural changes in HT29 cells; **(B)** large number of AVs in gPC (scale bar: 2 µm); **(C)** large late-stage AV in gO (scale bar: 5 µm); **(D)** disorganized nucleus with chromatin condensation (scale bar: 2 µm); **(E)** multivesicular body in gP (scale bar: 2 µm). K, control, non-treated HT29 cells; O, ODN2088; P, picropodophyllin; C, chloroquine; Kg, genomic DNA control; g, genomic DNA; arrows, autophagic vacuoles; MVB, multivesicular body; PM, plasma membrane; N, nucleus; M, mitochondrion; the presence of stars indicates significant differences from K (*p* < 0.05).

Thus, the presence of autophagy was observed in each group of HT29 cells, but to a varying degree. The representative microstructural changes together with the numerical data can be seen in Figure 7.

## Discussion

In our study, we attempted to answer how IGF1R inhibition modulates the effect of tumor-derived self-DNA on TLR9 signaling and autophagy response by examining the metabolic activity and proliferation of HT29 colon cancer cells.

First, we determined the effect of self-DNA-induced TLR9 signaling modulation on HT29 cell survival. The term “cell-free DNA” refers to all non-encapsulated DNA sequences in the blood stream. A portion of cfDNA is produced by tumor cells through apoptosis, necrosis, or active secretion [34, 35]. In addition to its role in the field of cancer diagnostics, cfDNA could influence the immune response, or promote tumorigenesis and “genometastasis” [36]. These biological effects can be triggered by signaling pathways activated by the interplay of cfDNA with certain cell receptors (including TLRs) or by increasing the transcriptional levels of several genes in an interaction similar to that observed with DNA aptamers [37].

HT29 cells constitutively express TLR9 mRNA [22]. The basal TLR9 gene expression in these cells is low, whereas TLR9 expression is increased by incubation with CpG-ODN or tumorous self-DNA [22, 38]. To support this, in our current experiment, we also detected increased TLR9 gene expression in HT29 cells treated with gDNA as compared to the control, non-treated HT29 group.

Regarding cell survival, we found that gDNA treatment with or without ODN2088, picropodophyllin, or chloroquine altered the metabolic activity and proliferation of HT29 cells to varying degrees. Interestingly, in the case of incubation with only gDNA, this was due to a decrease in the expression level of all examined genes (except for TLR9), while in the case of a combination of gDNA with inhibitors (i.e., O, C, and PC), an increase in gene expression was observed. Partly, this could be explained by the fact that the gDNA-triggered TLR9 signaling pathway may exhibit both survival-promoting and inhibitory effects [25, 39–43]. Blocking TLR9 signaling with ODN2088 increased cellular metabolic activity but did not significantly alter cell proliferation. gDNA treatment alone resulted in a notable decrease in cell proliferation. When ODN2088 was added to cells treated with gDNA, it had a tendency to counteract gDNA’s antiproliferative effect. This could be due to the differences in gene expression levels of central TLR signaling molecules, such as MAPK, PI3K, or NF-κB, which could play key roles [44].

**FIGURE 8:**
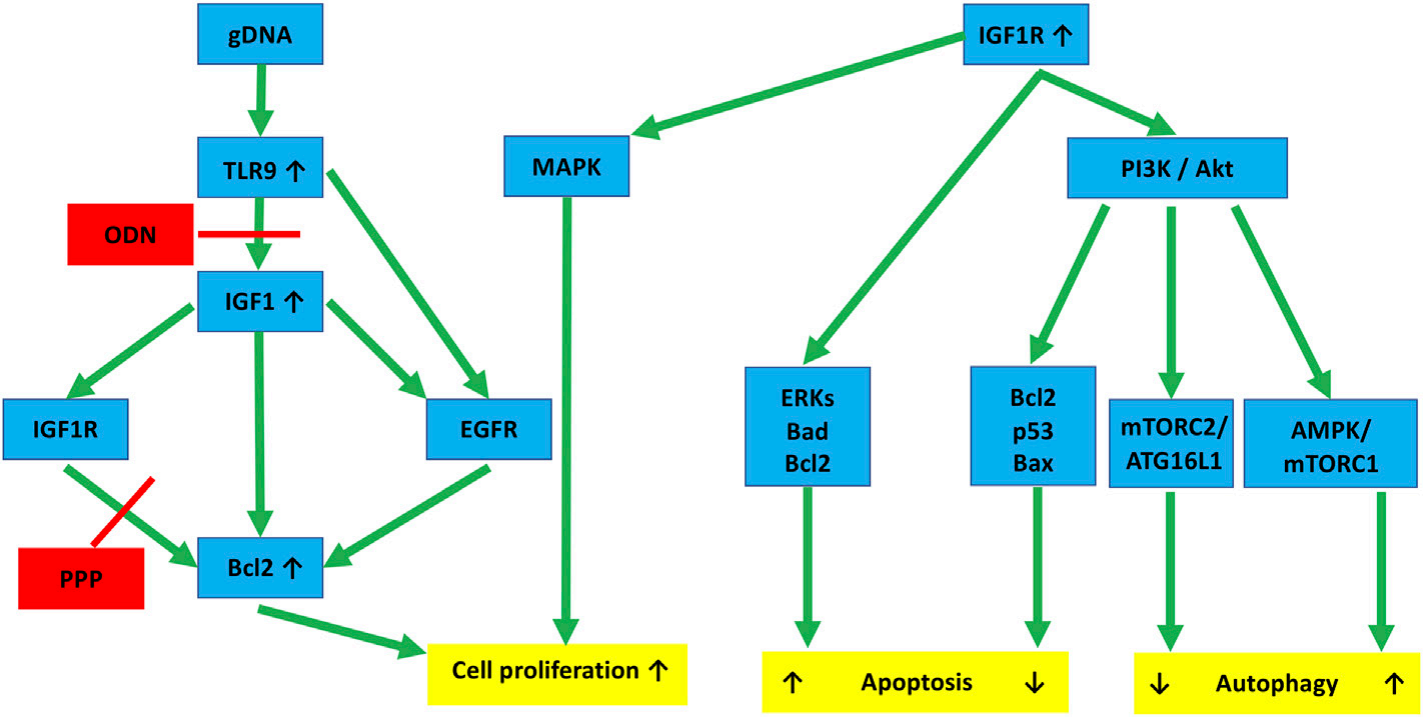
Hypothesized molecular links connecting IGF1R and TLR9 signaling to autophagy and cell proliferation in HT29 cancer cells. TLR9 binding of gDNA through IGF1 and IGF1R activation promotes cell division by enhancing Bcl2. ODN and PPP may inhibit this, but the inhibitory effect can be counteracted by EGFR cross-activation. IGF1R activation via the PI3K/Akt pathway affects autophagy. If it is through the AMPK/mTORC1 pathway, it is a stimulant. If it is through the mTORC2/ATG16L1 pathway, it is primarily inhibitory. Similarly, IGF1R inhibits apoptosis via the Akt-Bcl2-p53-Bax proteins, whereas the Erk-Bad-Bcl2 pathway tends to stimulate it. The final effects are always context-dependent. Red lines, inhibitory effect; gDNA, genomic self-DNA; TLR9, Toll-like receptor 9; ODN, oligodeoxynucleic acid 2088; IGF1, insulin-like growth factor 1; IGF1R, insulin-like growth factor 1 receptor; EGFR, epidermal growth factor receptor; PPP, picropodophyllin; Bcl2, B-cell lymphoma 2; MAPK, Mitogen-activated protein kinase; PI3K, Phosphoinositide 3-kinase; Akt, Ak strain transforming; Bax, BCL2 associated X; AMPK, AMP-activated protein kinase; mTOR, Mechanistic/mammalian target of rapamycin; mTORC1/2, mTOR complex 1/2; ERKs, extracellular signal-regulated kinases; ATG16L1, Autophagy Related 16 Like 1; Bad, BCL2-associated agonist of cell death.

In the following steps, the effect of changes in the interaction of IGF1R and TLR9 signaling pathways on HT29 cell survival was investigated. We observed that IGF1R inhibition *per se* increased the metabolic activity of HT29 cells, but did not significantly affect proliferation. gDNA treatment (and its combination with O, P, C, and PC treatments) significantly reduced cell division. However, gDNA with the combined inhibition of IGF1R and TLR9 signaling (i.e., gOP) abolished suppression of cell proliferation. In the background of this, a notable discrepancy is that the down-regulation of autophagy and apoptosis-related genes was observed, but Bcl2 was overexpressed. The anti-apoptotic and autophagy suppressor effects of Bcl2 overexpression are also reflected in the low level of AVs and increased number of cell divisions found in the gOP treatment group. Bcl-2 regulates programmed cell death during development and tissue repair, and it can have oncogenic abilities by inhibiting cell death rather than promoting cell proliferation [45]. TLR9 activation by CpG-ODN can increase the expression of IGF1 in intestinal epithelial cells [23], and intracellular IGF1 induces Bcl2 expression via IGF1R and epidermal growth factor receptor (EGFR) pathways [45]. According to these results, gDNA together with picropodophyllin could prevent Bcl2 overexpression. However, by adding ODN2088, Bcl2 became overexpressed, presumably via the IGF1R/EGFR or the TLR9/EGFR signaling cross-talk [45, 46]. Hence, the combined use of tumorous self-DNA and IGF1R inhibitors may display therapeutic (i.e., anti-proliferative) potential. Nevertheless, concomitant TLR9 inhibition may counteract this beneficial effect (Figure 8).

We also examined how the interaction of TLR9 and IGF1R signaling affects autophagy and HT29 cell proliferation. Autophagy can be triggered by CpG-ODNs in tumor cell lines (e.g., colon, breast, and prostate cancers) in a TLR9-dependent manner [47]. TLRs and autophagy may be linked by altered glyceraldehyde-3-phosphate dehydrogenase gene expression as well as the generation of reactive oxygen species [48, 49]. There are several shared features between TLR9 and autophagy pathways, such as their effects on cell survival and death, their interactions in endosomes, the positive effect of class III PI3K on their signaling, or their common inhibitors (e.g., chloroquine, 3-methyladenine, bafilomycin A1) [47]. We recently demonstrated a close relationship between TLR9 signaling and autophagy response, with remarkable effects on survival in HT29 cells treated with modified (i.e., hypermethylated or fragmented) self-DNA [25].

In this study, the use of IGF1R inhibition reduced autophagy and also mitigated the pro-autophagic effects of gDNA treatment and TLR9 signaling inhibition. However, concomitant use of chloroquine acted against picropodophyllin, i.e., increased autophagy.

Previous studies have shown that cellular autophagy can be mediated by PI3K/Akt inactivation and consequent AMPK/mTOR downregulation [50, 51]. We found that gDNA treatment caused the downregulation of these genes, which finally resulted in a reduction in the inhibition of autophagy initiation.

IGF1R activation can activate the PI3K/Akt and MAPK pathways, which are mediated by IRS1 and IRS2 phosphorylation after ligand binding [52, 53]. The PI3K/Akt pathway activates the mTOR pathway, which directs protein synthesis and cell growth via downstream effectors [53]. IGF1R can inhibit AMPK activity via Akt1, which phosphorylates an AMPK inhibitory site [54] (Figure 8). Picropodophyllin has been recently discovered as a potent inducer of autophagic flux that acts on-target [13].

In our experiment, by adding picropodophyllin to gDNA-treated cells, the relatively smaller decreases in PI3K, Akt, AMPK, and mTOR gene expressions resulted in an inhibitory effect on autophagy initiation, finally leading to a lower number of AVs. On the contrary, pharmacological inhibition of TLR9 resulted in the accumulation of AVs (as compared to gP), suggesting that ODN2088 decreased the anti-autophagic effect of gP combination. Since TLR9 sustains autophagic flux [55], this phenomenon is also understandable.

Chloroquine in itself has been found to inhibit cell growth by blocking autophagy in its late stage and inducing mitochondrion-mediated apoptosis [56]. While the gP combination had anti-autophagic effects, the addition of chloroquine (i.e., gPC) resulted in significantly increased autophagy as well as decreased cell proliferation. Based on these, we hypothesize that the complex balance of inhibitory and promoting factors of autophagy contributes greatly to the development of the ultimate intensity and final biological effect of the process. Regarding HT29 cell proliferation and autophagy, the different effects of P, O, and C individual and combined treatments also highlight that in addition to the TLR9-IGF1R-Bcl2 molecular link, the IGF1R-related and unrelated autophagy machinery is also “Janus-faced” in terms of its effect on cell proliferation.

In this study, we also investigated the impact of IGF1R and autophagy inhibition on the HT29 stem-like phenotype. The presence of CD133-positive cells accompanied by low cell proliferation activity and intense autophagy in the inhibitor-treated groups (i.e., gP, gC, gO, and gPC) calls attention to a potentially serious therapeutic consequence of IGF1R and/or autophagy-inhibition. In several tumor tissues, unbalanced IGF1R signaling can promote cancer cell proliferation and activate cancer reprogramming [2, 57]. Recently, IGF1R has been shown to facilitate epithelial-mesenchymal transition and cancer stem cell properties via Akt activation [58, 59]. In addition, autophagy is known to assist stem cell maintenance in several cell types [60]. In the case of IGF1R inhibition, the simultaneously induced protective autophagy could promote cell proliferation and suppress apoptosis. Thus, *via* autophagy, it antagonizes its own original biological effects on cells [11]. By combining IGF1R inhibition with autophagy disruptive agents, autophagy can be blocked, which can lead to suppressing cancer cell proliferation and enhancing apoptosis [11]. Because CD133-positive HT29 cells were only scattered in cell smears and could not be differentiated in TEM sections, we cannot tell how autophagy develops in CD133-positive HT29 stem-like cells. We only know that the overall autophagy flux in HT29 treatment groups is favorable for the appearance of the CD133 phenotype. However, the assessment of autophagic flux within CD133-positive HT29 stem-like cells would definitely be worth examining in the future.

From our experiment, it is possible that autophagy induced by different combinations of gDNA and the used inhibitors is unable to save HT29 cells from death. Furthermore, it could force some CD133-positive stem-like HT29 cells to promote survival. Supporting this hypothesis, it was found that curcumin promoted proliferation and autophagic survival of colon cancer stem cells [61]. This finding suggests the survival benefit from autophagy, permitting the long-term persistence of CRC stem cells [62].

By using TEM, we examined the relationship between IGF1R inhibition and autophagy to ultrastructural changes. In the case of gP-treated HT29 cells, multivesicular bodies (MVBs) were detected. There are at least three possible reasons for this phenomenon. The first is that receptors (such as the receptor tyrosine kinase IGF1R) can be quickly recycled back from early endosomes to the plasma membrane in a process called “back fusion.” During the maturation of the early endosome, its biochemical composition changes with increasing luminal acidification. Finally intraluminal vesicles, or MVBs are formed [57]. At this level, IGF1R can be delivered for recycling. The second is that MVBs containing endocytosed IGF1R can fuse with the plasma membrane and then release their content as “exosomes.” IGF1R has been reported to be released by cells in this way [2, 58, 59]. Thirdly, it has been recently demonstrated that in the absence of stromal cells, MVB-like small extracellular vesicle complexes can be released by HT29 cells [60].

In our study, the observed ultrastructural alterations call attention to the role of autophagy in cell protection or even in promoting cell death. In the gP cell group where the presence of MVBs was detected in addition to autophagic vacuoles, the expression of autophagy-related proteins (Beclin1, ATG16L1, LC3B) was increased as compared to control, non-treated cells. This result suggests that autolysosomal degradation is also likely to be present following the formation of amphisomes via the interconnection of autophagosome and multivesicular body pathways [61]. The amphisome functions as a prelysosomal compartment where the endocytic and autophagic pathways meet [62, 63]. The contents of amphisomes could have multiple fates, such as extracellular release or lysosomal degradation. Both exosome biogenesis and autophagy display pivotal roles in maintaining cellular homeostasis and enhancing stress tolerance [64–68]. Influencing these functions for cancer cells may allow the identification of realistic therapeutic targets.

## Conclusion

In summary, the combination of tumorous self-DNA treatment with ODN2088, picropodophyllin, or chloroquine alters the metabolic activity and proliferation of HT29 cells to varying degrees. Inhibition of TLR9 signaling adversely affects the influence of self-DNA treatment on cell proliferation. The concomitant use of tumor-derived self-DNA and IGF1R inhibitors displays anti-proliferative potential. However, parallel TLR9 signaling inhibition negatively changes this beneficial effect. We found that the complex balance of inhibitory and promotional factors in autophagy greatly contributes to the characteristics of its final intensity and biological outcomes. The different effects of picropodophyllin, ODN2088, and chloroquine alone or in combination on HT29 cell proliferation and autophagy suggest that in addition to the TLR9-IGF1R-Bcl2 molecular linkage, the IGF1R-associated and non-IGF1R-associated autophagy machinery is “Janus-faced” in terms of its effect on cell proliferation. Based on our results, it is also possible that autophagy, induced by different combinations of tumorous self-DNA and inhibitors is not sufficient to rescue HT29 cells from irreversible death, but may result in the survival of some CD133-positive stem-like cancer cells, which could promote CRC recurrence. The ultrastructural changes we observed also support the context-dependent role of IGF1R inhibition and autophagy on cell survival and proliferation.

The creation of new types of combined IGF1R, autophagy, and/or TLR9 signaling inhibitors would play a significant role in the development of more personalized anti-tumor therapies. However, our present experiments should be tested in additional TLR9-expressing cell lines. Further research is also mandatory to investigate the biological effects of modified self-DNA fragments, since methylation status or fragment length may also affect the experimental results.

## Data Availability

The original contributions presented in the study are included in the article/Supplementary Material, further inquiries can be directed to the corresponding author.

## Supplementary Material

Supplementary Figure S1 Regarding immunocytochemistries, the results of the Tukey HSD test and the adjusted *p*-values of the comparisons of each treatment group can be seen (*p* < 0.05 represents statistical significance).
