# Supplementary figures and images for: Survival of HT29 Cancer Cells Is Affected by IGF1R Inhibition via Modulation of Self-DNA-Triggered TLR9 Signaling and the Autophagy Response
Source: Pathol Oncol Res. 2022 May 16;28:1610322. doi: 10.3389/pore.2022.1610322 (PMC9148969; doi:10.3389/pore.2022.1610322)

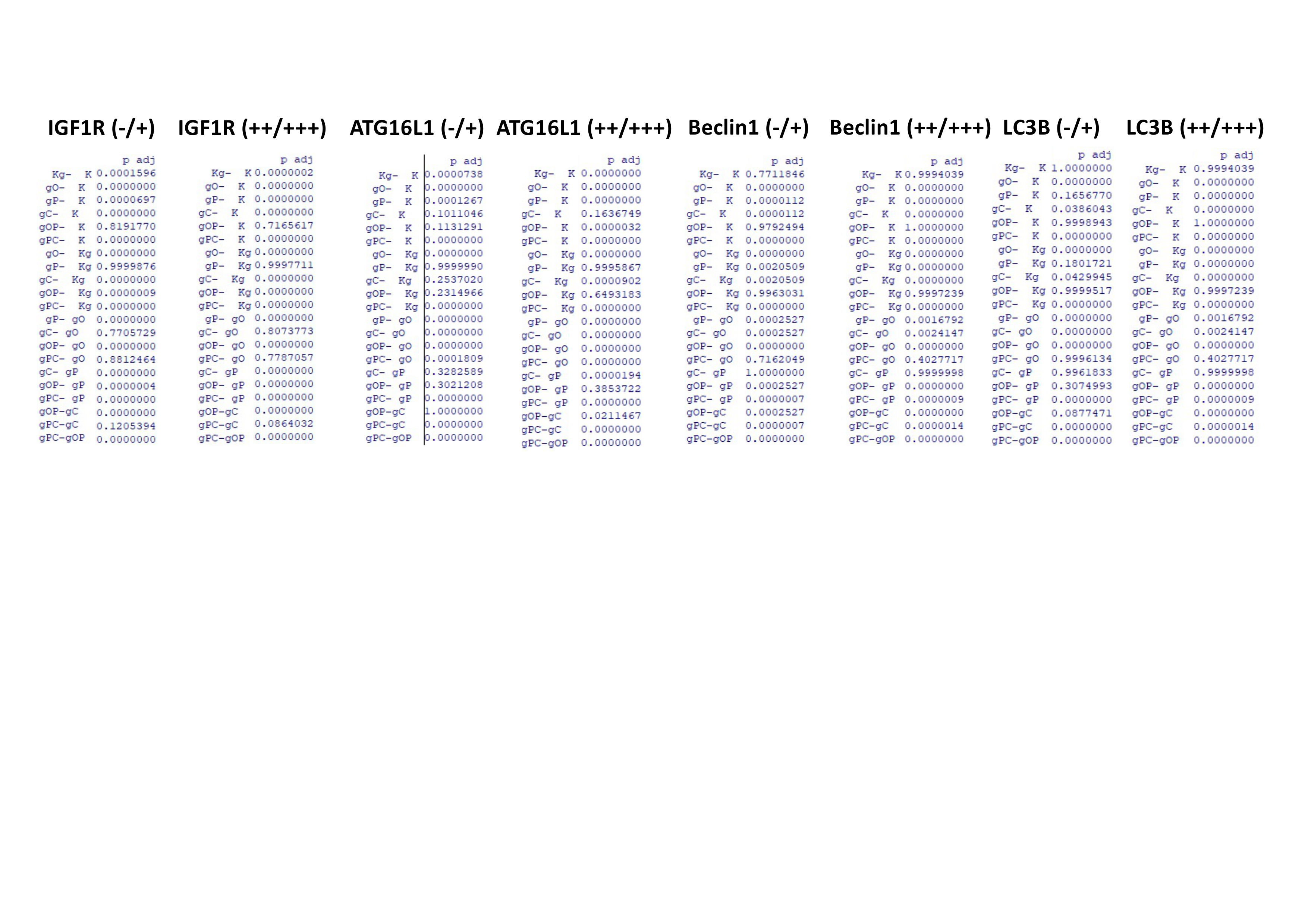

Supplement: Supplementary file 1 [file Image1.TIF]
